# Head and neck cancer: pathogenesis and targeted therapy

**DOI:** 10.1002/mco2.702

**Published:** 2024-08-21

**Authors:** Yan Liu, Nannan Zhang, Yi Wen, Jiaolin Wen

**Affiliations:** ^1^ Frontiers Medical Center Tianfu Jincheng Laboratory Chengdu China; ^2^ National Facility for Translational Medicine (Sichuan) West China Hospital of Sichuan University Chengdu China; ^3^ National Center for Birth Defect Monitoring Key Laboratory of Birth Defects and Related Diseases of Women and Children Ministry of Education West China Second University Hospital Sichuan University Chengdu China; ^4^ State Key Laboratory of Biotherapy West China Hospital of Sichuan University Chengdu China

**Keywords:** clinical strategies, HNC, immunotherapy, pathogenesis, targeted therapy

## Abstract

Head and neck cancer (HNC) is a highly aggressive type of tumor characterized by delayed diagnosis, recurrence, metastasis, relapse, and drug resistance. The occurrence of HNC were associated with smoking, alcohol abuse (or both), human papillomavirus infection, and complex genetic and epigenetic predisposition. Currently, surgery and radiotherapy are the standard treatments for most patients with early‐stage HNC. For recurrent or metastatic (R/M) HNC, the first‐line treatment is platinum‐based chemotherapy combined with the antiepidermal growth factor receptor drug cetuximab, when resurgery and radiation therapy are not an option. However, curing HNC remains challenging, especially in cases with metastasis. In this review, we summarize the pathogenesis of HNC, including genetic and epigenetic changes, abnormal signaling pathways, and immune regulation mechanisms, along with all potential therapeutic strategies such as molecular targeted therapy, immunotherapy, gene therapy, epigenetic modifications, and combination therapies. Recent preclinical and clinical studies that may offer therapeutic strategies for future research on HNC are also discussed. Additionally, new targets and treatment methods, including antibody–drug conjugates, photodynamic therapy, radionuclide therapy, and mRNA vaccines, have shown promising results in clinical trials, offering new prospects for the treatment of HNC.

## Graphical abstract

Current clinical treatment of HNC. Tobacco, alcohol, viral infection, genomic changes, head and neck X‐ray exposure, and poor oral hygiene are the main causes of HNC. At present, the preferred treatment for most HNC patients is postoperative combined with chemotherapy or/and radiotherapy. Emerging therapeutic strategies mainly include immune checkpoint inhibitors, costimulatory agonists, antigen vaccines, OV therapy, CAR‐T, multireceptor targeting MAb and small molecule inhibitors, gene therapy, natural medicine therapy, and combination therapy (created with BioRender.com).

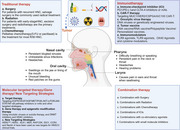

## 1 INTRODUCTION

Head and neck cancer (HNC) is a frequently diagnosed malignancy with 946,456 new cases and 482,001 deaths reported in 2022 according to GLOBOCAN.^1^ About 90% of these cases are head and neck squamous cell carcinoma (HNSCC).^2^, ^3^ The incidence of HNSCC is projected to increase by 40% by 2040 reaching nearly 600,000 new cases annually.^4^ Diagnoses are occurring at younger ages, largely due to the rising infection rates of human papillomavirus (HPV), especially HPV‐16 and HPV‐18. Besides HPV infection, studies have shown that tobacco use, chronic heavy alcohol consumption, oral sex, mechanical irritation, radiation exposure, and various occupational exposures can also contribute to the increased incidence of HNC.^5^, ^6^ Additionally, Epstein–Barr virus (EBV) and hepatitis B virus are other pathogenic factors for HNC and are considered biomarkers for nasopharyngeal carcinoma.^7^, ^8^ However, with the implementation of HPV vaccination, decreased smoking rates, enhanced promotion of safe sex practices, and early precision diagnosis, it is anticipated that the incidence of HNC will decline by 2060.^3^, ^6^, ^9^

Although the incidence of HNC might decrease in the future, the 5‐year survival rates of HNC remain unsatisfactory. In the United States from 2012 to 2018, the 5‐year survival rates of tumors in oral cavity and pharynx were 68 and 61% for tumors in the larynx.^10^ The prognosis of hypopharyngeal tumor is even poorer, with a 5‐year survival rate of only 25%.^11^, ^12^ The poor outcomes of HNC are often due to late diagnosis, recurrence, metastasis, and drug resistance.^13^, ^14^ Generally, 60−65% HNC patients can be cured by surgery and/or radiotherapy (RT) if detected early. However, patients with locoregional recurrence, metastatic diseases, or second primaries may require chemoradiation.^15^ Patients undergoing chemoradiotherapy may experience significant side effects and a reduced quality of life.^16^ Targeted and immune‐based therapies, either alone or in combination with conventional treatments, offer promising new strategies for HNC. For example, combining afatinib with cisplatin enhances anticancer activity in HNSCC, inhibiting tumor growth and spread.^17^ The combination of pembrolizumab with chemotherapeutics is considered a first‐line therapy for R/M HNSCC.^18^

Despite significant improvements in the combined therapeutic efficacy of HNC over the past few decades, further research is required to identify the most appropriate therapeutic strategies, given the genetic heterogeneity among HNC patients and the complexity of the disease. This paper reviews recent advances in understanding the molecular pathogenesis of HNC, including changes in signal pathways, immune microenvironment suppression, and the impact of genetic and epigenetic alterations on disease progression. It also discusses various treatment methods such as targeted therapy, immunotherapy, gene therapy, or combination therapy, and summarizes the drugs currently being researched or available on the market. The goal is to identify the most effective treatment plans from existing therapeutic strategies and provide a theoretical foundation for the clinical management of HNC.

## 2 PATHOGENESIS OF HNC

As a tumor with high heterogeneity, late diagnosis and high recurrence rate, the pathogenesis of HNC is extremely complex, involving many aspects, including signaling pathway abnormalities, tumor microenvironment (TME) inhibition, genomic changes, epigenetic changes,^19^ and so on. Additionally, risk factors like HPV infection, smoking, alcohol abuse and poor oral health play significant roles in the onset and progression of HNC.^20^ We will discuss the factors related to the occurrence and progression of HNC, providing a foundation for its clinical treatment.

### 2.1 HPV infection in HNC

HPV infection is a major factor contributing to the rising incidence of HNC patients.^21^ Among all the chronic HPV infections in HNC patients, nearly 85% are caused by HPV16 or HPV18. HPV can cause changes in oncogenes, including amplification, rearrangement, deletion, and translocation, and it induces the expansion and expression of E5/E6/E7 proteins,^22^, ^23^, ^24^ leading to the initiation and progression of HNC.^25^

E6 and E7 proteins are particularly crucial in HPV‐positive HNC.^25^ E6 interacts with the retinoblastoma protein (RB), reducing inhibition of E2F transcription factors, and degrades the tumor suppressor protein p53 through a ubiquitin‐dependent pathway. The disrupts normal cell cycle regulation and promotes HNC proliferation and growth.^26^ Additionally, E6 upregulates proapoptotic proteins (BAK and BAX) by suppressing P300 and cyclic adenosine monophosphate response element binding protein, or by disrupting apoptotic signaling pathways, thus affecting apoptosis.^26^ E6 also enhances tumor immune evasion by inhibiting interferon regulatory factor 3, Toll‐like receptors 9 (TLR9)/CD289, and by activating cyclin‐dependent kinase 2.^27^, ^28^ E7 also binds to RB, promoting its proteasome degradation, preventing apoptosis, senescence, and cell cycle arrest in host cells, and thereby encouraging viral production and tumor development.^29^, ^30^ Expression of p16 inhibits pRb phosphorylation, which is correlated with HPV infection status and survival outcomes in HNSCC.^31^ In addition, E6 participate in cell division and immortalization by upregulating TERT.^32^ E5 proteins also play significant role in the development of HNSCC through activation of the epidermal growth factor receptor (EGFR) signaling pathway.^33^

Beyond pathogenesis, HPV infection status can influence the therapeutic efficacy for HNC. The genetic, epigenetic, and TME differences between HPV‐positive and HPV‐negative HNC, as well as treatment variations, will be discussed in detail in the following sections.^34^

HPV infection alone is not enough for tumor development, other risk factors like EBV infection and smoking and alcohol consumption are necessary.^21^, ^35^ EBV is another significant factor in the occurrence and development of HNSCC.^36^, ^37^ It promotes HNSCC by manipulating various cell signaling pathways to protect infected cells from the immune system.^38^ Rahman et al.^39^ found that almost 12% of patients with HNSCC have coinfection with HPV and EBV, and Deng et al.^40^ has revealed that these two viruses synergistically promote tumor occurrence and development. HPV can facilitate EBV entry into epithelial cells by upregulating integrin and CD21, help establish EBV's incubation period and the activation of its cleavage cycle, and promote local immune escape, leading to secondary EBV infection. Together, HPV and EBV promote the development of HNSCC.^41^

HPV vaccines targeting E6/E7 have showed the ability to generate virus‐specific cytotoxic T lymphocytes (CTLs), but these vaccines have not demonstrated significant antitumor effects.^42^ Interestingly, recent RNA‐Seq and whole genome sequencing (WGS) studies on HPV‐positive HNC revealed that most of these cancers express E2 instead of E6. This could explain the limited efficacy of current therapeutic vaccines for HPV‐positive cancers and indicate E1/E2 may be better targets for future therapies.^43^

### 2.2 The pathogenesis signaling pathways in HNC

**FIGURE 1:**
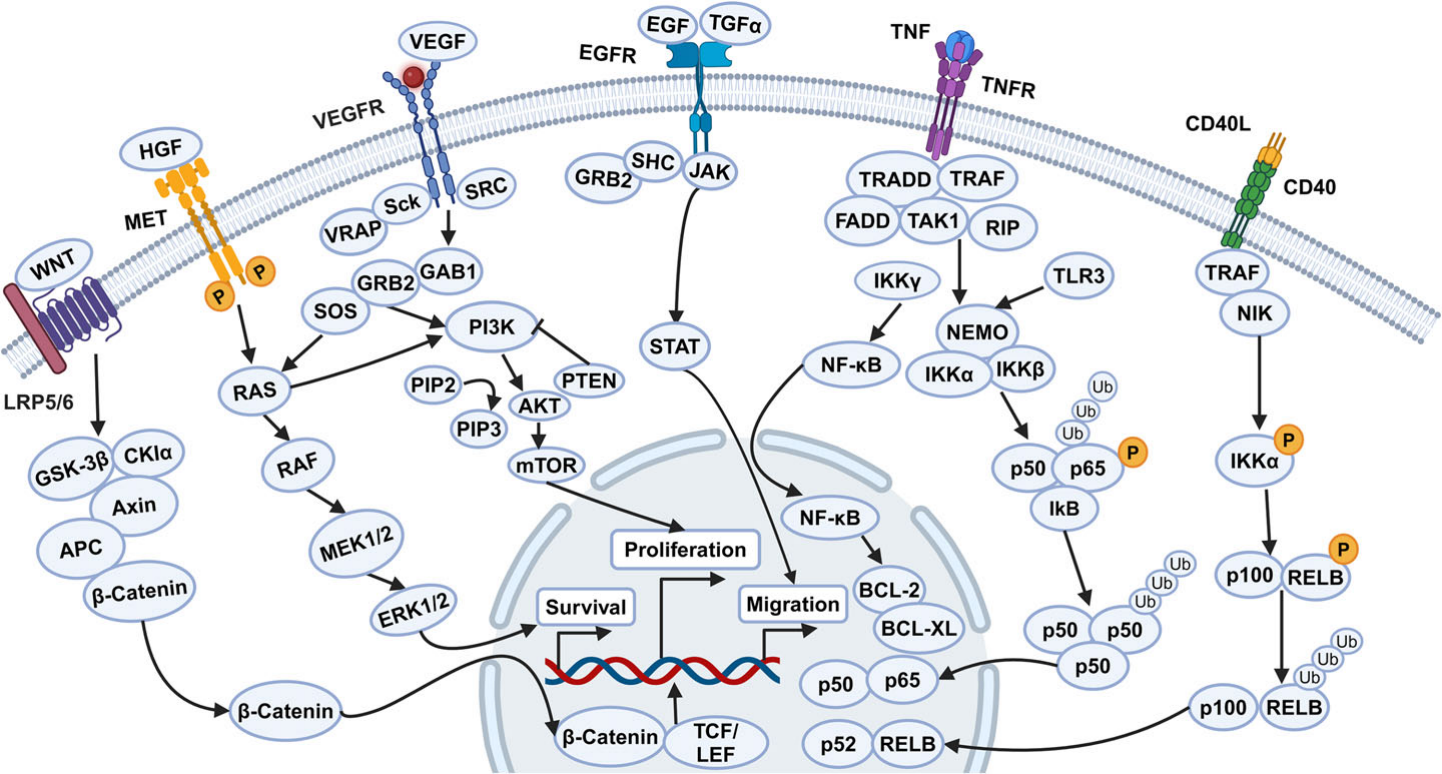
Signaling pathway alterations in HNC. The signaling pathway alterations in HNC are complex and diverse, involving multiple genes and proteins. Two critical signaling pathways in HNC are the EGFR pathway and the HGF/MET pathway. Activation of EGFR and HGF/MET signaling can lead to increased cell growth, resistance to apoptosis, and the promotion of tumorigenesis and tumor progression. Another significant pathway is the PI3K/AKT/mTOR pathway, where activation results in enhanced cell growth, survival, and angiogenesis, further driving tumorigenesis and progression. Other important signaling pathways include JAK/STAT, NF‐κB, VEGF/VEGFR, and WNT/β‐catenin. Dysregulation of these pathways contributes to cell proliferation, apoptosis, immune evasion, and metastasis, thereby promoting tumor development and progression. LRP5/6, low‐density lipoprotein‐related receptors 5 and 6; TCF/LEF, T cell factor/lymphoid enhancer factor family (created with BioRender.com).

HNC is characterized by a complex signaling network involving numerous genes and proteins. In HNC, there is an overexpression of four key cell surface receptors: EGFR, VEGFR, HER2, and MET. These receptors drive downstream pathways that are crucial for cell proliferation, apoptosis, immune escape and metastasis (Figure 1). Here, we will focus on the PI3K/AKT/mTOR, JAK/STAT, NF‐κB, HGF/MET, and TP53/RB pathways.

#### 2.2.1 PI3K–AKT–mTOR signaling

Activated PI3K/AKT/mTOR signaling is associated with radiation therapy and cellular drug resistance.^44^, ^45^, ^46^ In HNSCC, this pathway is often overactivated due to the amplification or mutation of PIK3CA and the amplification or overexpression of AKT. PIK3CA amplification and PTEN inactivation are common in HPV‐positive tumors. The inactivation or deletion of PTEN weaken its lipid phosphatase activity, reducing its inhibition of PI3K pathway. This leads to upregulation of AKT and an increased rate of cell proliferation, which in turn promotes the expansion and metastasis of HNSCC.^2^ Mutation or amplification of PIK3CA abnormally activate this pathway, accelerating the onset and progression of HNSCC. Several drugs targeting this pathway are currently in clinical trials.^46^, ^47^, ^48^ Dual targeting of mTOR and EGFR is recommended to overcome drug resistance.

#### 2.2.2 JAK/STAT signaling

The JAK/STAT signaling pathway is abnormally activated in HNC due to mutations in various oncogenes affecting receptors, downstream mediators, and related transcription factors.^49^, ^50^, ^51^ Research has shown that the abnormal activation of STAT3 and STAT5 is related to cell proliferation, angiogenesis, immune escape, metastasis, and resistance to treatment.^51^, ^52^ In several types of HNSCC cells, STAT3 signaling has been reported to be activated by the RTK Family, alpha‐7 nicotine receptors, the interleukin family (IL‐6, IL‐10, and IL22 receptors), erythropoietin receptors, TLRs, GPCRs, TGF‐α activation, and circular RNA FAT1 (circFAT1).^2^, ^51^, ^53^, ^54^ Phosphorylated STAT3 upregulates the expression of cyclins and cytokines, thereby promoting the development of HNSCC. Jia et al.^54^ discovered that circFAT1 can promote phosphorylation of STAT3, and knocking down circFAT1 demonstrated antitumor effects both in vitro and in vivo. Phosphorylated STAT3 can also lead to the inactivation of Caspase‐3 and Caspase‐9, inhibiting the apoptosis of tumor cells. Additionally, the abnormal activation of the JAK/STAT pathway can promote the differentiation of Th1 and Th2 cells, affecting the type and intensity of the immune response. In HNSCC, the abnormal activation of JAK/STAT pathway can also promote the expression of PD‐1 and matrix metalloproteinase 9 (MMP‐9), creating an immunosuppressive state and inducing the degradation of the extracellular matrix (ECM), which promotes the progression and metastasis of HNSCC. Currently, Numerous studies have showed that targeting this pathway can inhibit the growth of HNSCC, including compounds or small molecule inhibitors like FLLL12, AG490, AZD1480,^55^ and so on. JAK/STAT is emerging as a promising pathway for drug development in HNSCC.^52^

#### 2.2.3 NF‐κB signaling

Several studies have shown that the upregulation and abnormal activation of NF‐κB family plays significant role in HNSCC.^56^, ^57^, ^58^ Among the pathogenic factors of HNSCC, cigarette smoke can lead to phosphorylation and degradation of IκBα, activating the NF‐κB pathway. In addition, the NF‐κB pathway can interact with the PI3K/AKT, EGFR, and JAK/STAT pathways. Overexpression of EGFR, as upstream signal, can also activate NF‐κB signaling, contributing to the production of IL‐8 and VEGF, which promote the proliferation and metastasis of HNSCC. Researches indicates that NF‐κB can enhance the production and spread of reactive oxygen species (ROS) by inducing enzymes such as nitric oxide synthase (iNOS), causing DNA damage and carcinogenic damage to surrounding cells.^59^ Carcinogens and ROS‐related genetic and epigenetic alterations collectively affect upstream signal transduction, leading to the abnormal activation of IKK and NF‐κB, which in turn contributes to the development of HNSCC.

#### 2.2.4 HGF/MET signaling

Research data have showed that HGF and MET proteins were overexpressed in HNSCC.^60^ These proteins influence the onset and progression of HNSCC through multiple pathways. Notably, MET and EGFR share a downstream signaling pathway, indicating that these receptors collaborate to enhance tumor cell proliferation, movement, invasion, malformation, angiogenesis, metastasis, and resistance to chemotherapy (CT). In addition, the c‐Met pathway can upregulate WNT/β‐catenin and ERK/c‐Fos pathways, further driving tumor proliferation and migration.^61^

#### 2.2.5 TP53/RB signaling

TP53 mutations are the most frequent among the TSGs in HNSCC.^62^, ^63^ The primary causes of p53 protein dysfunction in HNSCC include gene mutation and HPV infection. In HPV‐positive HNSCC patients, p53 is inactivated following degradation induced by E6 viral protein binding. While in HPV‐negative HNSCC patients, TP53 gene mutations are the primary cause of p53 protein dysfunction.^64^, ^65^ In addition, TP53 inactivation can lead to reduced phosphorylation of the RB protein, increasing the activity of the E2F transcription factor. Pathways like WNT/β‐catenin pathway also interact with the TP53/RB pathway, contributing to HNSCC mechanisms. Recent studies have proposed p53‐based therapeutic strategies, such as introducing WT‐TP53 into HNSCC cells to restore p53 activity or using compounds to reactivate WT p53 function in cells with mutant p53. Strategies also include using compounds to target carcinogenic mutant p53 for degradation or hitting downstream pathways of mutant p53, leading to synthesis lethality.^62^, ^65^, ^66^ Although few p53‐targeted drugs have advanced to late‐stage clinical trials and none have been marketed. TP53 mutation status holds potential for prognosis evaluation and predicting chemoradiotherapy efficacy in HNSCC patients.

### 2.3 Immune regulation mechanism

The TME in HNC includes various immune cells, which participate in immune escape, suppression and response. This section discusses these immune cells and the mechanisms of immune escape.

#### 2.3.1 Immune microenvironment in HNC

**FIGURE 2:**
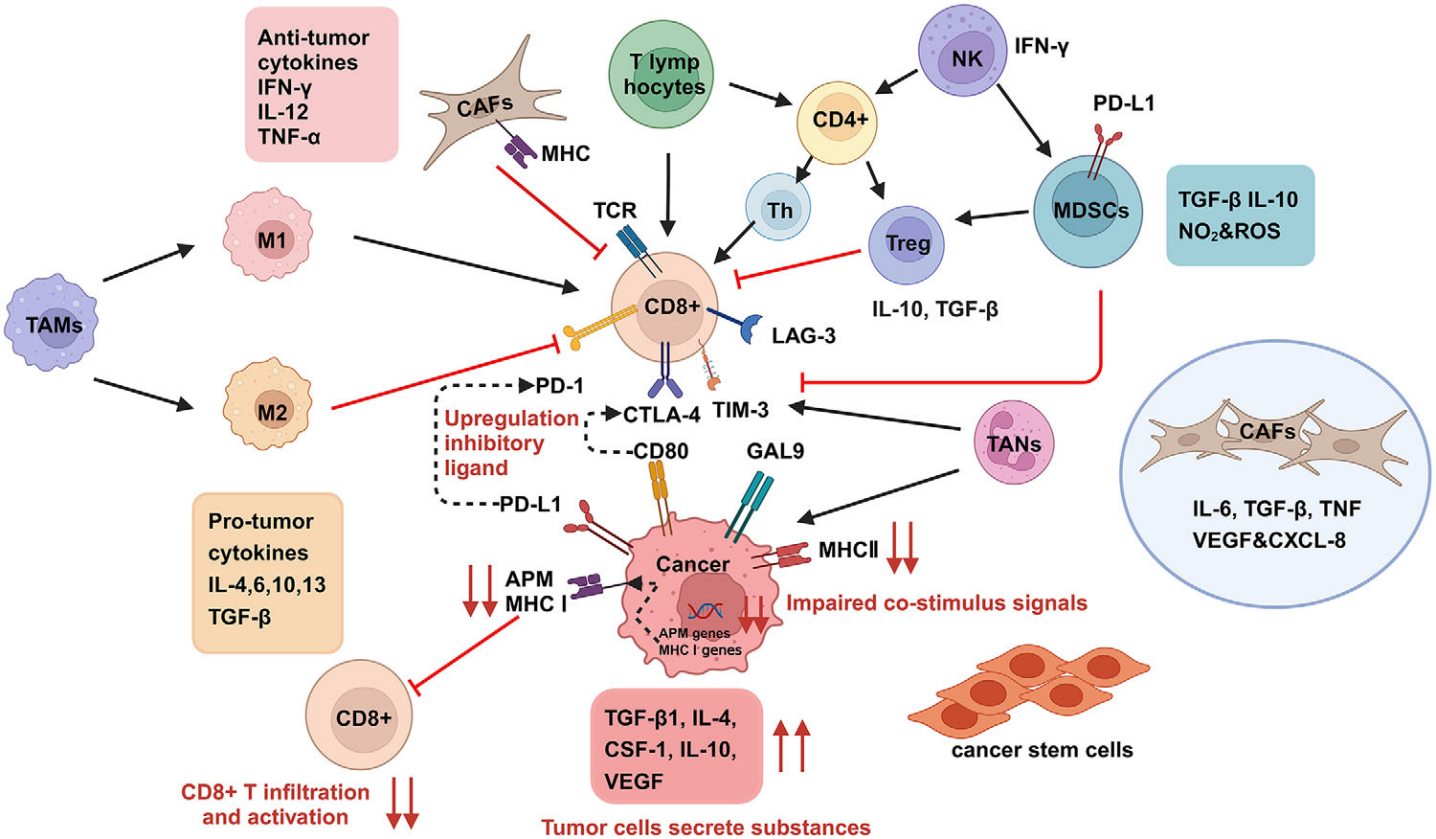
Immune microenvironment in HNC. Immune cells in HNC include TAMs, T lymphocytes, CAFs, NK cells, Treg cells, MDSCs, and TANs. Immune escape plays a crucial role in the tumor microenvironment. Tumor cells can overexpress ligands such as PD‐L1 and CD80 on their surfaces, which bind to receptors like PD‐1 and CTLA‐4 on activated T cells, allowing the tumor cells to evade recognition and attack by the immune system, thereby achieving immune escape. Tumors can also evade CD8^+^ T cell recognition by downregulating the expression levels of MHC I and APM genes, inhibiting CD8^+^ T cell infiltration and activation. Additionally, cancer cells and the surrounding stromal cells can continuously secrete various substances that facilitate immune escape and drug resistance, including VEGF, CSF‐1, IL‐4, and IL‐10. These substances not only directly inhibit T cell function but also promote the accumulation of inhibitory immune cells. TAMs, tumor‐associated macrophages; MDSCs, myeloid‐derived suppressor cells; CAFs, cancer‐associated fibroblasts; TANs, tumor‐associated neutrophils; ROS, reactive oxygen species (created with BioRender.com).

The TME in HNC consists of both nontumor cells and ECM proteins. The cellular components include genetically altered stromal cells, blood and lymph vascular cells, and infiltrating immune cells. Noncellular components include ECM proteins and physicochemical parameters. HNSCC is heterogeneous with two different TMEs based on HPV infection status. HPV‐negative HNSCC patients have lower CD8^+^ T cells infiltration and higher levels of monocytes, macrophages, NK T cells and neutrophils.^67^, ^68^ HPV‐positive HNSCC patients exhibit higher expression of B cells, plasma cells, Th1 and Th2 CD4^+^ T cells, CD8^+^ T cells, Treg cells, dendritic cells (DC), and CD56^dim^ NK cells.^69^ Tumor‐ablating M1 macrophages and protumoral M2 macrophages coexist in the TME. A high M1/M2 macrophages ratio is associated with better prognosis in HPV‐positive tumors.^70^ In addition, cancer‐associated fibroblasts (CAFs) produce HGF, VEGF, TGF‐β, IL6, CXCL1, CXCL12, and PD‐L2, promoting tumor growth and antagonizing antitumor immune responses by recruiting suppressive immune cells.^71^, ^72^ Recent single‐cell analyses have identified premetastatic cell subpopulations related to AXL and AURK pathways^73^ (Figure 2).

HPV infection interacts complexly with the TME of HNSCC.^74^ It can affect TAMs, myeloid cells, DC, and NK cells, facilitating immune escape. HPV infection also downregulates the expression of HLA, impairing T cells recognition of HNSCC and promoting immune escape. What is more, HPV increases the expression of IL‐6, IL‐8, and TGF‐β, enhancing tumor cell proliferation, migration, and invasion. It also influences VEGF expression, promoting tumor blood vessel formation and expansion.

On the other hands, HGF/c‐Met plays a role in TME alterations, where HGF is paracrinally produced by CAFs in the surrounding TME.^61^, ^75^ The activation of HGF and Met promotes HNSCC cell proliferation, growth, spread, and metastasis. HGF influences radio and chemoresistance through glycolytic pathways, leading to drug resistance in HNSCC patients.^76^ Regarding tumor immunity, HGF/MET signaling converts M1 macrophages to M2‐like phenotypes, with M2 macrophages promoting HNSCC cell growth.^77^ HGF/Met signaling also increases lactic acid secretion via glycolysis, inhibiting human CTL proliferation and activity, and triggering tumor recurrence and metastasis. HGF can upregulate PD‐L1 expression, aiding immune evasion.

#### 2.3.2 Immune escape in HNC

The resistant and recurrent nature of HNC suggests that an immune escape mechanism is at play. Recent studies indicate that HNC cancer cells evade immune detection primarily through four mechanisms: upregulation of inhibitory ligands, engagement of immune checkpoints, impaired costimulatory signals, and secretion of substances from tumor cells (Figure 2).

##### 2.3.2.1 Upregulation of inhibitory ligand

The immune system contains inhibitory checkpoints such as CTLA‐4, PD‐1, TIM‐3, LAG‐3, TIGIT, B7‐H3, and B7‐H4, which act as “brakes” to regulate T cell activity. TIM‐3 interacts with several ligands, including GAL‐9, CEACAM1, phosphatidylserine (PS), and HMGB1 protein. The ligand for LAG‐3 is FGL1, while TIGIT interacts with CD96 and CD226.^78^ Tumor cells expressing PD‐L1 and PD‐L2 can activate PD‐1 on T cells, inhibiting T cell immune responses, promoting apoptosis of lymph node antigen‐specific T cells, and reducing apoptosis of Tregs and macrophages. Additionally, PD‐L1 from activated T cells can bind to PD‐1 on macrophages, promoting M2 polarization. Similarly, CTLA‐4 on cancer‐activated T cells can bind to the B7 ligand on HNC cells, leading to T cell inactivation.^79^ In HNSCC, the TME is immunosuppressive, and the upregulation of inhibitory ligands hinders the immune system's ability to eliminate tumor cells, resulting in tumor progression.

##### 2.3.2.2 Engagement of immune checkpoints

Studies show that PD‐L1 is expressed in about half of HNC cases.^80^ Other immune checkpoints, such as CD276 and CD44, are also overexpressed in HNSCC. These proteins decrease the activity of CD8^+^ T cells by binding to corresponding proteins on T cells, thereby inhibiting the anticancer abilities of immune cells.^81^ Wang et al.^81^ established mouse models of HNSCC with in vivo lineage tracing of tumor stem cells, confirming that CD276 is elevated in tumor stem cells and protects them from T cell attacks. Prince et al.^82^ identified CD44 as a cancer stem cell marker for HNSCC, finding significant increases in CD44 levels and associations with tumorigenesis, radiation resistance, CT resistance, and an increased immunosuppressive phenotype of HNSCC.^83^, ^84^, ^85^

##### 2.3.2.3 Impaired costimulus signals

Cancer cells can evade recognition by CD8^+^ T cells by downregulating the MHC‐I/II, making antigens unrecognizable. It is revealed that the expression of MHC‐I class molecules is reduced due to the downregulation of human leukocyte antigen genes in HNSCC.^86^ A latest research showed that MHC‐I associated antigen presentation machinery (APM) plays a crucial role in activating tumor‐killing effector T cells, contributing to the low response rate of HNSCC to immune checkpoint inhibitors (ICIs).^87^

##### 2.3.2.4 Secreted substances from tumor cells

HNSCC and its surrounding matrix continuously secrete substances like VEGF, CSF‐1, IL‐4, IL‐10, and TGF‐β, which facilitate tumor immune escape and drug resistance. These substances not only directly inhibit T cell function but also promote the aggregation of inhibitory immune cells.^88^

### 2.4 Pathogenic genetic factors

In HNC, genetic and epigenetic factors combine to influence gene expression, leading to changes in cell signaling pathways that regulate tumor growth, DNA repair, antiapoptosis, angiogenesis, resistance to external factors, and epithelial mesenchymal transformation.^89^

**FIGURE 3:**
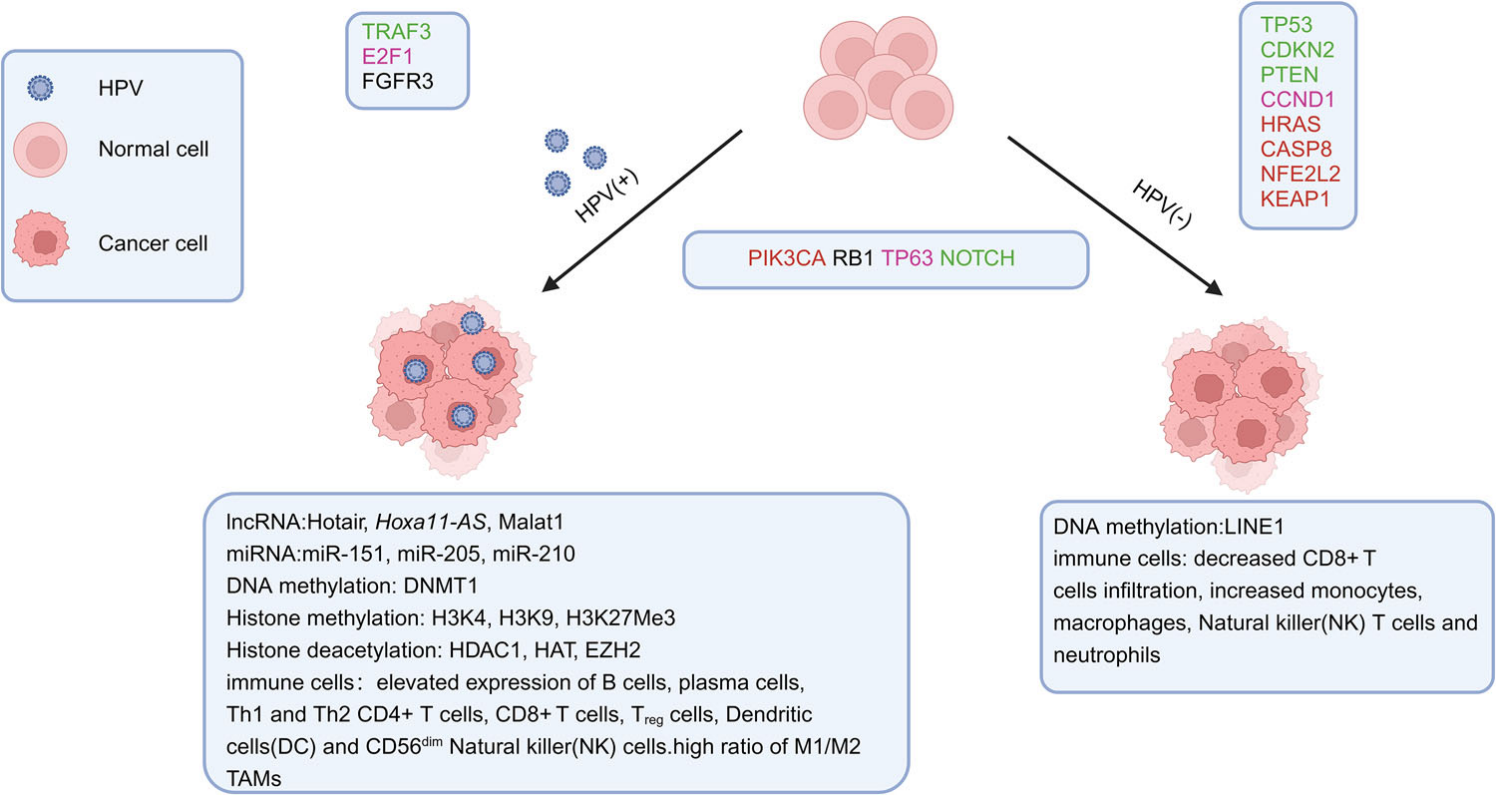
HPV infection, genetic, epigenetic, and TME changes in HNC. HPV infection is a significant risk factor for the development of HNC. HNC is divided into two main subgroups: HPV‐positive and HPV‐negative. Common mutations in HPV‐positive and HPV‐negative HNSCC include PIK3CA, RB1, TP63, and NOTCH. Additionally, more than 70% of HPV‐positive HNSCC patients have genetic mutations, primarily in the FGFR3, E2F1, and TRAF3 genes. In contrast, HPV‐negative HNSCC exhibits a significant diversity of genetic alterations, mainly involving TP53, CDKN2A, PTEN, CCND1, HRAS, CASP8, NFE2L2, and KEAP1 genes. Many noncoding RNAs involved in various biological processes in HNSCC are also altered, such as lncRNA (Hotair，HOXA11‐AS，Malat1) and miRNA (miR‐151, miR‐210, miR‐205) in HPV‐positive HNSCC. Epigenetic factors play a crucial role in the occurrence and development of HNSCC, with epigenetic modifications primarily including DNA methylation and posttranslational covalent modifications of histones (methylation and acetylation). Key epigenetic modifications include DMNT1, HDAC1, EZH2, and H3K27Me3 in HPV‐positive HNSCC, and LINE1 in HPV‐negative HNSCC. LINE1, long interspersed nuclear elements 1; NFE2L2, nuclear factor erythroid‐derived 2‐like 2; KEAP1, Kelch‐like ECH‐associated protein 1; Hotair，HOX antisense intergenic RNA; *HOXA11‐AS*, homeobox (HOX) A11 antisense lncRNA; Malat1, metastasis‐associated lung adenocarcinoma transcript 1 (created with BioRender.com).

Researches on molecular biomarkers of HNSCC indicates frequent chromosomal losses and genetic instability. Losses of 9p21, 3p21, 17p13, 11q13, 13q21, 14q32, 6p, 8, 4q27, and 10q23 are marked at different stages of progression.^90^ Tumor suppressor genes (TSGs) such as CDKN2A and TP53 are involved in early stages of HNSCC,^91^ while PTEN is implicated in later stages, rather than oncogene mutation. Key TSGs also include FAT1, NOTCH1, KMT2D, NSD1, and TGFBR2.^92^ The only oncogene frequently mutated in HNSCC is PIK3CA, with a mutation rate of 14%.^93^, ^94^ Mutations in CDKN2A, TP53, NFE2L2, and KEAP1 are restricted to HPV‐negative tumors. Conversely, frequent losses of TRAF3, NSD1, FAT1, NOTCH1, and SMAD4, amplifications of E2F1 and genes encoding EGFR, HER2, and FGFR1, and alterations in PIK3CA, PTEN, FBXW7, and KRAS occur in HPV‐positive tumors^91^ (Figure 3). A new study in 2023 identified seven differential genes (CCR4, WDFY4, VCAM1, LYZ, VSIG4, XIRP1, and CMKLR1) as crucial in the TME.^95^ Recent research also showed that genes including ACTN1 and TNFAIP2 play significant roles in tumor progression and CT resistance, suggesting ACTN1 as a novel target in HNSCC.^96^, ^97^ Another study revealed that the metabolic biomarker PYGL promotes progression, metastasis, and CT resistance of HNSCC through the GSH/ROS/p53 pathway.^98^

In addition, data from The Cancer Genome Atlas^22^ showed that smoking‐associated HNSCC exhibits widespread gene mutations and continual copy number changes. Functional studies of these genes indicate their critical roles in the development and progression of HNSCC, and targeting these potential genes may be significant for HNSCC treatment.^99^

### 2.5 Epigenetic mechanism in HNC

Epigenetic modifications, unlike gene mutations, alter the phenotype without changing the DNA sequence. These modifications are heritable and reversible.^100^ Epigenetic alterations include DNA methylation,^101^, ^102^, ^103^ histones modifications, chromatin remodeling, and noncoding RNA (ncRNA) activity (Figure 3).^104^, ^105^

#### 2.5.1 DNA methylation

DNA methylation is a common epigenetic modification involving the covalent addition of methyl groups to the five position of cytosine (5‐MC).^100^ Laytragoon‐Lewin et al.^106^ found DNA methylation in multiple TSGs in HNSCC and linked abnormal methylation to shorter survival after standard therapy. Zhou et al.^103^ identified and confirmed the diagnostic value of two hypermethylated and two hypomethylated genes as prognostic biomarkers in HNSCC. Pan et al.^107^ subsequently identified six more methylated genes serving as prognostic markers. The E6 protein inactivates TP53 by promoting the DNMT1 promoter, affecting the methylation of several genes, such as CDKN2A, RASSF1, CCNA1, Cadherin family genes, ITGA4, TIMP3, ELMO1, MEI1, and LINE1. CDKN2A is hypomethylated in HPV‐positive HNC cases, while RASSF1 is hypomethylated in normal head and neck cells. Genes like CCNA1, Cadherin family genes, ITGA4, and ELMO1 are hypermethylated in HNC, and LINE1 is hypomethylated in HPV‐negative tumors. Further research is required to establish these epigenetic changes as HNC biomarkers.^108^ In addition, one study has shown that the risk of HPV‐HNC can be predicted by detecting DNA methylation levels in HPV late genes.^109^

#### 2.5.2 Histone modification

Aside from DNA methylation, histone modification is another significant predictive epigenetic mechanism in HNSCC.^110^ Histones, composed of N‐terminal and globular C‐terminal domains, undergo posttranslational modifications primarily at the N‐terminal. Acetylation, deacetylation, and methylation of histones are closely linked to transcriptional activation and gene expression. Chen et al.^111^ demonstrated that acetylation of lysine 27 on histone H3 (H3K27ac) at the promoter of long ncRNAs (lncRNA) plac2 leads to upregulation of plac2 and activation of the WNT/β‐catenin pathway, impacting HNSCC progression. John et al.^89^ found that paracrine‐induced histone modification increased expression of Bmi‐1, a transcriptional suppressor related to HNSCC invasiveness. What is more, Ma et al.^112^ discovered that a new substance LncMX1‐215 directly binds H3K27 acetylase GCN5, blocking H3K27 acetylation and inhibiting the proliferation and transfer ability of HNSCC both in vitro and in vivo. In addition, E7 also regulates HADC1 and EZH2.^113^

#### 2.5.3 Noncoding RNAs

ncRNAs play a crucial role in various biological processes and are altered in HNSCC tumor tissues. NcRNAs, which include small ncRNAs (miRNAs, siRNAs, and piRNAs) and lncRNAs, are vital for cell homeostasis, development, and differentiation.

lncRNAs can bind to RNAs and proteins, regulating gene expression and infecting cell proliferation, survival, and metastasis. They interact with pathways like JAK/STAT3, TGF‐/Smad, and WNT/β‐catenin. Key HNSCC‐related lncRNAs include HOTAIR, HOXA11‐AS, MALAT1, ANRIL, and H19. HOTAIR, for example, recruits EZH2 to catalyze H3K27 trimethylation, inhibiting TSGs, and also acts on miR‐206 to activate the PI3K/AKT pathway, promoting HNSCC development^114^

miRNAs prevent translation or downregulate mRNA of target genes, participating in HNC proliferation, differentiation, development, and apoptosis. They also regulate pathways like WNT/β‐catenin, PTEN/AKT/mTOR, JAK/STAT, and TGF‐β. Overexpression of carcinogenic miRNAs promotes the occurrence and development of HNSCC. While downregulation of tumor suppressor miRNAs like miR‐99a is associated with increased invasion, cell cycle progression, cell proliferation, and clonal formation of HNSCC.^104^ Overexpression of miR‐107, miR‐151, miR‐182, miR‐361, miR‐324‐5p or low expression of miR‐492, miR‐20b is correlated with disease‐free survival and overall survival (OS) in OC/OPSCC patients. In HPV‐positive HNSCC, miR‐151 is upregulated while miR‐210 and miR‐205 are downregulated.^104^

Other dysregulated miRNAs in HNSCC include miR‐99a, HSA‐miR‐29c‐3p, miR‐128, miR‐375, miR‐32‐5p, miR‐26a/b, miR‐376c, miR‐876‐5p, miR‐200a, miR‐93, miR‐205‐5p, miR‐124‐3p, miR‐29s, miR‐92a‐3p, miR‐150, miR‐203, miR‐545, miR‐532‐3p, miR‐204‐5p, miR‐200, miR‐26a, and miR‐145 have been summarized.^115^ miR‐375 is correlated with metastasis and shorter survival, which has promised to be a biomarker of poor prognosis in HNSCC.^116^ In addition, downregulation of hsa‐let‐7d and hsa‐miR‐205 in HNSCC tumors, showing potential as a poor prognosis biomarker of HNSCC.^117^ Microarray analysis revealed 20 miRNAs^118^ were highly expressed in HNSCC samples, including miR‐372, miR‐320, miR‐21, miR‐34a, miR‐200c, miR‐223, hsa‐miR‐32‐5p, miR‐654‐5p, miR‐187, miR‐510, miR‐626, miR‐107, miR‐103, miR‐24, miR‐450a, miR‐122‐5p, and hsa‐miR‐375,^115^, ^119^ and most of these can be considered as diagnostic and prognostic biomarkers in HNC.

In addition, miRNAs in HNC regulate drug resistance through pathways involving the cell cycle, apoptosis, DNA repair, EMT, CSCs, and drug efflux pumps.^120^, ^121^

## 3 TARGETED THERAPY

**FIGURE 4:**
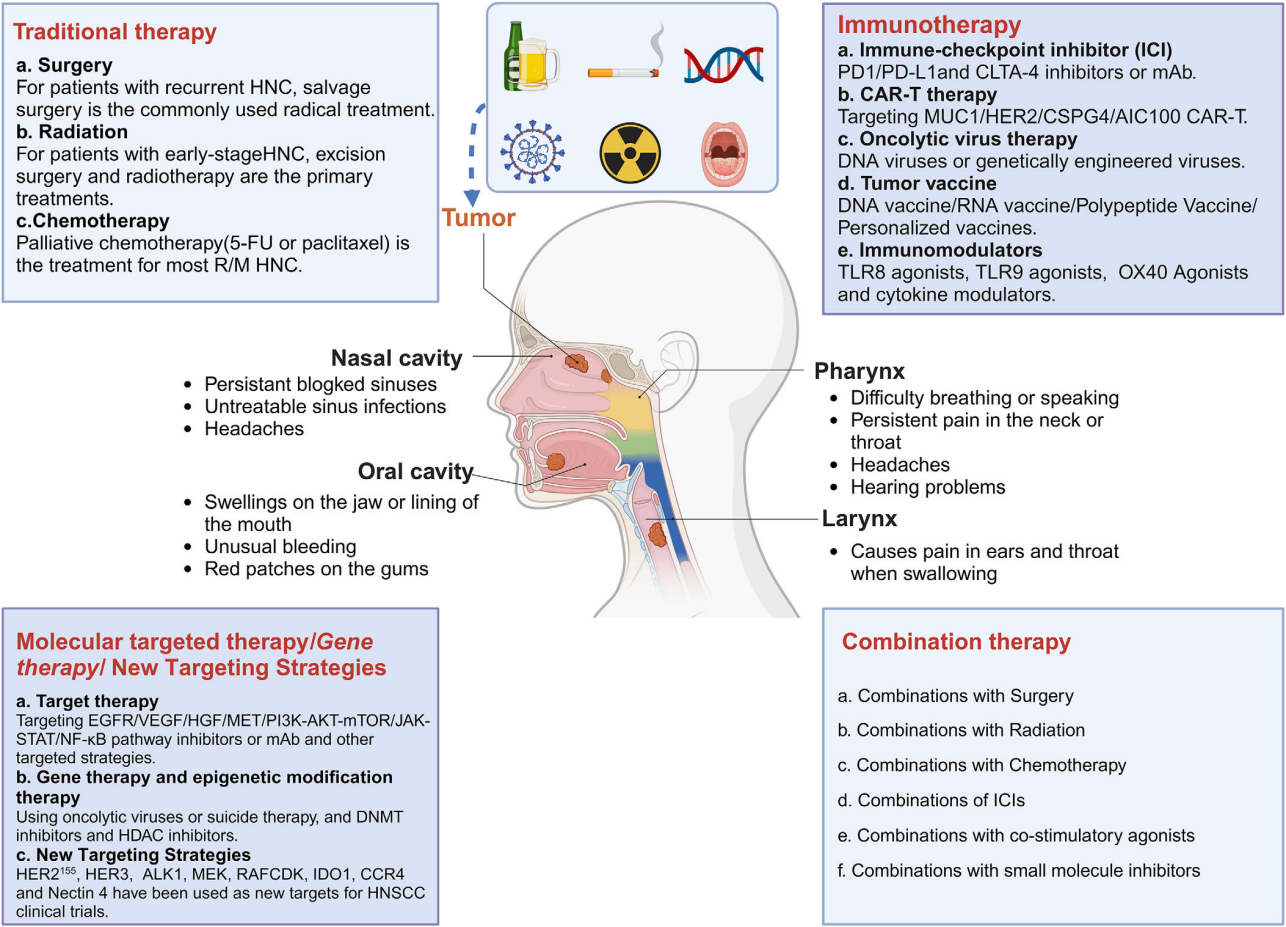
Current clinical treatments of HNC. Tobacco, alcohol, viral infection, genomic changes, head and neck X‐ray exposure, and poor oral hygiene are the main causes of HNC. At present, the preferred treatment for most HNC patients is postoperative combined with chemotherapy or radiotherapy. Emerging therapeutic strategies mainly include immune checkpoint inhibitors, costimulatory agonists, antigen vaccines, OV therapy, CAR‐T, multireceptor targeting MAb and small molecule inhibitors, gene therapy, natural medicine therapy, and combination therapy. CSPG4, chondroitin sulphate proteoglycan 4, IDO1, indoleamine2,3‐dioxygenase1 (created with BioRender.com).

Traditional treatments for HNC primarily include surgery, RT, and CT. Currently, surgery remains the cornerstone of HNC treatment except for nasopharynx cancer. However, as our understanding of the molecular characteristics and pathogenesis of HNC has deepened over the past decade, treatment strategies have evolved significantly. This evolution has introduced targeted therapy, immunotherapy,^122^, ^123^, ^124^, ^125^, ^126^, ^127^, ^128^ and gene therapy, either alone or in combination with traditional methods like surgery, RT, or CT^129^ (Figure 4).

By searching for the keywords “head and neck,” “HNC,” “head and neck squamous cell carcinoma,” “HNSCC,” “nasopharyngeal carcinoma,” “SCC of the nasopharynx,” and “NPC” on PubMed, Web of Science, and the National Cancer Institute (NCI) databases: we summarized the therapeutic drugs currently in use or under clinical investigation worldwide. To date, 20 drugs have been brought to market, including ICIs, antibodies targeting EGFR, reactive oxygen activators, and CT agents. Three single‐molecule therapies, cetuximab, pembrolizumab, and nivolumab, have been approved for treatment of R/M HNSCC following platinum resistance. Cetuximab in combination with RT has been approved for locally advanced HNSCC (Table 1). Furthermore, we searched ClinicalTrials.gov for drugs related to HNC and summarized the latest research progress of various drugs in clinical trials. These include molecular targeted therapy drugs, immunotherapy drugs, gene therapy drugs, epigenetic modification therapy drugs, and emerging therapy drugs (Table 2).

**TABLE 1:** Drugs approved worldwide for the treatment of HNCs.

Drug(s)	Target(s)	Indication	First approved country/region
Pembrolizumab	PD‐1	HNSCC, R/M HNSCC, head and neck neoplasms	America
Nivolumab	PD‐1	Head and neck neoplasms, HNSCC	America
Toripalimab	PD‐1	NPC	China
Tislelizumab	PD‐1	NPC	China
Camrelizumab	PD‐1	NPC, recurrent NPC	China
Nimotuzumab	EGFR	Head and neck neoplasms, NPC	India
Cetuximab	EGFR	Head and neck neoplasms, HNSCC	America
ASP‐1929	EGFR	Unresectable locally advanced or recurrent HNC	Japan
Docetaxel	Tubulin	Head and neck neoplasms	Japan
Fluorouracil	TYMS	Head and neck neoplasms	Japan
Nitrocaphane	DNA	Nasopharyngeal carcinoma	China
Carboplatin	DNA	Head and neck neoplasms, HNSCC	Japan
Cisplatin	DNA	Head and neck neoplasms	Japan
Cyclophosphamide	DNA	Head and neck neoplasms	China
Technetium Tc‐99 M Tilmanocept	CD206	OSCC	America
Hydroxycarbamide	RNR	HNSCC, HNC	America
Methotrexate sodium	DHFR	HNSCC	America
Recombinant human adenovirus type 5	p53	NPC	China
Bleomycin A5 hydrochloride	–	NPC	China
Temoporfin	ROS	HNSCC	European

Abbreviations: HNSCC, head and neck squamous cell carcinoma; HNC, head and neck cancer; NPC, nasopharyngeal carcinoma; OSCC, oral squamous cell carcinoma.

**TABLE 2:** Clinical trials of HNCs.

Classifications	Interventions	Target (s)	Clinical trials	Phase	Enrollment	Indication
Molecular targeted therapy						
EGFR pathway	Nimotuzumab	EGFR	NCT00957086	III	710 (Estimated)	HNSCC
	Zalutumumab	EGFR	NCT00496652	III	619 (Actual)	HNC
	Afatinib	EGFR	NCT01427478	III	134 (Actual)	HNSCC
VEGF pathway	Bevacizumab	VEGF	NCT05063552	II/III	430 (Estimated)	R/M HNSCC, R/M LSCC, R/M Lip and oral cavity carcinoma, R/M PSCC, R/M SSCC, R/M NSCC, metastatic HSCC
	Sorafenib	VEGFR	NCT00494182	II	48 (Actual)	R/M HNSCC, R/M HSCC, R/M lip and oral cavity carcinoma
	Sunitinib	VEGFR	NCT00387335	II	22 (Actual)	Metastatic SNC with occult primary, recurrent LSCC, recurrent NSCC, recurrent OSCC
	Cabozantinib	VEGFR, MET, AMT, AXL	NCT03468218	II	36 (Actual)	R/M HNSCC, PSSCC, recurrent HSCC, recurrent LSCC, recurrent OSCC
HGF/MET pathway	Ficlatuzumab	HGF	NCT06064877	III	410 (Estimated)	R/M HNSCC
PI3K/AKT/mTOR pathway	Buparlisib	PI3K	NCT04338399	III	483 (Estimated)	HNC
	Everolimus	mTOR	NCT00942734	II	49 (Actual)	HNC
	Ipatasertib	AKT	NCT05172258	II	52 (Estimated)	R/M HNSCC, R/M LSCC, R/M OSCC, R/M HSCC
JAK/STAT pathway	Danvatirsen	STAT3	NCT02499328	I/II	340 (Actual)	Advanced solid tumors, metastatic HNSCC
	Ruxolitinib	JAK1/2	NCT03153982	II	16 (Actual)	HNSCC
New targeting pathways	Trastuzumab	HER2	NCT06130007	II	48 (Estimated)	OSCC
	Patritumab	HER3	NCT02633800	II	87 (Actual)	Head and neck neoplasms
Immunotherapy						
ICIs	Finotonlimab	PD‐1	NCT04146402	III	330 (Estimated)	HNSCC
	Durvalumab	PD‐L1	NCT02551159	III	823 (Actual)	HNSCC
	Atezolizumab	PD‐L1	NCT01810913	II/III	613 (Estimated)	p16INK4a‐negative OSCC, LSCC, HSCC, OCSCC
	Avelumab	PD‐L1	NCT02999087	III	707 (Actual)	HNSCC
	Ipilimumab	CLTA‐4	NCT03700905	III	276 (Estimated)	HNC
	Tremelimumab	CLTA‐4	NCT03624231	II	18 (Actual)	HNSCC
CAR‐T/NK	CAR T cells	MCU1	NCT05239143	I	100 (Estimated)	Nasopharyngeal cancer, HNSCC
		HER‐2	NCT03740256	I	45 (Estimated)	HNSCC, cancer of the salivary gland
		CSPG4	NCT06096038	I/II	33 (Estimated)	HNC
		LMP‐1	NCT02980315	I/II	20 (Estimated)	Nasopharyngeal neoplasms
	CAR‐NK cells	PD‐L1	NCT04847466	II	55 (Estimated)	Advanced HNSCC
Oncolytic virus	RP3/Herpesvirus	–	NCT05743270	II	130 (Estimated)	HNSCC, locally advanced HNSCC, recurrent HNSCC
Tumor vaccines	Anti‐MUC1 vaccine	MUC1	NCT02544880	I/II	16 (Actual)	HNC, HNSCC
Peptide‐based	p16(INK4a)	p16	NCT01462838	I/II	26 (Actual)	HPV‐induced cancers
	UCPVax vaccine	Telomerase	NCT03946358	I/II	47 (Estimated)	HNSCC
	IO102/IO103	IDO	NCT04445064	II	17 (Estimated)	HSCC, LSCC, OSCC, OCSCC
Nucleic acid‐based	ISA101 vaccine	HPV16 E6/E7	NCT02426892	II	33 (Actual)	Solid tumors
	MEDI0457	HPV16/18E6/E7	NCT03162224	II	35 (Actual)	HNC, HPV Associated HNSCC
Personalized cancer vaccines	VB10.NEO	–	NCT03548467	I/II	41 (Actual)	Locally advanced or metastatic solid tumors
	AlloVax	–	NCT01998542	II	12 (Actual)	HNC, HNSCC
Cell‐based vaccine	DC vaccine	p53	NCT00404339	I	17 (Actual)	HNC
Immunomodulators	Motolimod	TLR8	NCT02124850	I	18 (Actual)	HNSCC
	EMD 1201081	TLR9	NCT01040832	II	107 (Actual)	HNSCC
	IRX‐2	IL‐2	NCT00210470	II	27 (Actual)	HNSCC
Gene therapy						
Adenovirus‐carrying endostatin gene	E10A	VEGFR	NCT02630264	III	540 (Estimated)	Head and neck neoplasms
			NCT00634595	II	116 (Estimated)	HNSCC, NC
Recombinant human endostatin adenovirus	EDS01	VEGFR	NCT02283489	II	180 (Estimated)	Head and neck neoplasms
Epigenetic modification therapy						
DNMT1 inhibitors	Azacytidine	DNMT1	NCT05317000	I	50 (Estimated)	HNSCC
	Decitabine	DNMT1	NCT05265962	II	85 (Estimated)	ESCC
HDAC inhibitors	Romidepsin	HDAC	NCT00084682	II	14 (Actual)	Stage IV HSCC, Stage IV OCSCC, Stage IV OSCC
	Panobinostat	HDAC	NCT00670553	I	7 (Actual)	HNC
	CUDC‐101	HDAC	NCT01171924	I	47 (Actual)	HNC
Emerging therapies						
APP inhibitor	EGCG	APP + DYRK1A + α‐synuclein	NCT01116336	I	25 (Actual)	Head and neck neoplasms
CCR4 antagonist	FLX475	CCR4	NCT03674567	I/II	323 (Actual)	Advanced cancer
Cell INDUCTION	E7 TCR‐T cells, aldesleukin	HPV E7	NCT05639972	I/II	15 (Estimated)	HPV+ OSCC, OC
mRNA vaccine	BNT‐113	E6, HPV E7	NCT04534205	II	285 (Estimated)	Unresectable HNSCC, R/M HNC
Therapeutic vaccine	HB‐201	E6, HPV E7	NCT04630353	I	10 (Actual)	HPV 16+ confirmed OC
ADC	OBT076	CD205	NCT05930951	I	32 (Estimated)	Adenoid cystic carcinoma of HNC

Abbreviations: HNC, head and neck cancer; HNSCC, head and neck squamous cell carcinoma; HSCC, hypopharyngeal squamous cell carcinoma; LSCC, laryngeal squamous cell cancer; NC, nasopharyngeal carcinoma; NSCC, nasopharyngeal squamous cell carcinoma; OC, oropharynx cancer; OCSCC, oral cavity squamous cell carcinoma; OSCC, oral squamous cell carcinomas; PSSCC, paranasal sinus squamous cell carcinoma; PSCC, pharyngeal squamous cell carcinoma; SSCC, sinonasal squamous cell carcinoma; SNC, squamous neck cancer.

*Sources*: Data were obtained from ClinicalTrials.gov.

### 3.1 Molecular targeted therapy

Compared with the toxicity and drug resistance caused by traditional treatment methods, molecular targeted therapy offers higher selectivity and fewer adverse reactions.^130^ By targeting cell surface receptors such as EGFR, VEGFR, HER2, and MET, and their downstream signaling pathways, it is possible to inhibit HNSCC cell proliferation, invasion, and migration. Additionally, targeting epigenetic modification sites like histone acetylation/deacetylation or DNA methylation can inhibit HNSCC migration and proliferation.^131^

#### 3.1.1 Targeting cell surface receptors

Cell surface receptors like EGFR, VEGFR, HER2, and MET are overexpressed in HNC, driving tumor growth, cell migration, and metastasis.

(1) EGFR inhibitor

Monoclonal antibodies (McAbs) targeting EGFR include cetuximab, nimotuzumab, panitumumab, zalutumumab, duligotuzumab, and imgatuzumab. Tyrosine kinase inhibitors (TKIs) targeting EGFR include selective targeted TKIs (gefitinib, erotinib, sapitinib) and dual‐targeted TKIs (afatinib, lapatinib, dacomitinib).

Cetuximab was the first EGFR inhibitor approved for HNSCC in 2006, with monotherapy response rates for recurrent/metastatic (R/M) HNSCC around 10−15%.^132^ Cetuximab has shown increased efficacy when combined with cisplatin/carboplatin, fluorouracil, and paclitaxel in R/M HNSCC.^133^ Recent clinical trials combining cetuximab with various inhibitors have demonstrated durable activity and safety.^134^ However, cetuximab treatments can cause severe skin, mucosal, kidney, and gastrointestinal toxicity.^135^ New inhibitors like nimotuzumab, panitumumab, and zalutumumab have been developed with fewer side effects and higher EGFR affinity. Nimotuzumab combined with cisplatin/RT demonstrated good effects in a phase II study. Panitumab is being evaluated in several clinical trials for locally advanced HNSCC and R/M HNSCC (NCT00547157, NCT00500760, NCT00820248, and NCT00798655) or R/M HNSCC (NCT00454779). Another clinical trial showed that zalutumumab increased survival to 9.9 weeks from 8.4 weeks in platinum‐resistant R/M HNSCC patients.^2^ Duligotuzumab is a McAb double targeted to EGFR and HER3. Additional antibodies like milatuzumab, a humanized anti‐EGFR McAb designed to enhance antibody‐dependent cellular cytotoxicity, have shown promising efficacy for metastatic colorectal cancer with KRAS mutations.^136^

Several small molecule EGFR inhibitors are also used in HNSCC treatment. Gefitinib and lapatinib are the first‐generation oral EGFR‐TK inhibitors. Although gefitinib did not show significantly efficacy, it improved patients' quality of life compared with CT drugs for advanced HNSCC.^137^ When applied in combination with other therapies, gefitinib showed a good response rate and good survival (4‐year OS, 74%).^2^ Lapatinib was showed with the clinically activation in HPV‐positive patients (53 cases) and locally advanced HNSCC (56 cases).^138^ Afatinib a second‐generation irreversible EGFR/HER2 inhibitor,^139^ overcomes some resistance issues seen with first‐generation drug.

(2) VEGF/VEGFR inhibitors

Targeting VEGF/VEGFR can inhibit angiogenesis in HNSCC, which include VEGF inhibitors (bevacizumab, ramucirumab) and VEGFR inhibitors (for example, sorafenib, anlotinib, apatinib, donafenib, sunitinib, cabozatinib, and vandetanib). Bevacizumab was the first antiangiogenic agent approved by the United States Food and Drug Administration (US FDA) for cancer treatment.^140^, ^141^ Although bevacizumab has not been approved for HNSCC treatment, clinical studies have demonstrated its efficacy and safety in patients with HNSCC. Although bevacizumab alone or in combination has a positive effect, its adverse effects such as bleeding and sensitivity are still needed further studies. Sorafenib is a multikinase inhibitor, including BRAF, VEGFR1, and VEGFR‐2, which promotes apoptosis, slows angiogenesis, and inhibits tumor cell proliferation.^142^ Recent studies have shown that sorafenib can be used as a sensitizing agent to enhance the antitumor effect of chemoradiotherapy on HNSCC by downregulating ERCC‐1 and XRCC‐1 DNA repair proteins.^143^ Several clinical trials, including NCT00494182, NCT02035527, NCT00815295, NCT00939627, and NCT00096512, are evaluating sorafenib in combination with cisplatin, 5‐FU, docetaxel, or paclitaxel on R/M HNSCS; sunitinib and apatinib are also multitargeted inhibitors of VEGFR, PDGFR, and c‐kit. A recent preclinical study showed that Sunitinib inhibited the activation of MDSCs and enhanced antitumor immunity when combining with tametatin (an EZH2 inhibitor).^144^

(3) HGF/MET inhibitors

MET activation is associated with resistance to cetuximab in HNSCC. Current inhibitors targeting the HGF/MET pathway include c‐Met inhibitors (cabozantinib, stantinib, forvitinib) and HGF inhibitor (ficlatuzumab). Cabozantinib is also a TKI of VEGFR‐1/2/3/AXL/c‐Met/TAM receptor.^145^ Cabozantinib also has immunomodulatory effects, including inhibition of AXL19 and MER signaling. A recent clinical trial (NCT03468281) showed that cabozantinib and pembrolizumab had improved PFS and OS in R/M HNSCC, and an overall clinical benefit rate of 91%.^146^ The clinical trial results of carbozantinib combined with cetuximab (NCT03667482) showed a certain efficacy of carbozantinib in CTX‐resistant R/M HNSCC patients.^145^ However, stanitinib and forvitinib have not demonstrated promising clinical efficacy in R/M HNSCC.

Ficlatuzumab is a humanized anti‐HGF IgG1 mAb. Multiple clinical trials (NCT06064877, NCT02277197, NCT03422536) compared the combined efficacy of ficlatuzumab and cetuximab in OS in patients with R/M HNSCC. The results show that this well‐tolerated combination regimen has good antitumor activity in cetuximab‐resistant advanced HNSCC and better efficacy in HPV‐negative HNSCC patients.^147^, ^148^

#### 3.1.2 Targeting PI3K–AKT–mTOR

Abnormal activation of the PI3K/AKT/mTOR pathway is observed in 30.5% of HNSCC patients, and clinical trial results show that targeting PI3K/AKT/mTOR pathway‐related components can inhibit the metastasis, expansion, and deterioration of HNSCC.

Inhibitors targeting PI3K include generic Class I inhibitors (buparlisib, copanlisib) and only targeting PI3K P110α‐specific inhibitor (alpelisib). Buparlisib is a potent pan‐class I PI3K inhibitor and showed good efficacy in combination with paclitaxel for advanced HNSCC.^149^ A KURRENT‐HN study showed that more than 45% of patients with PI3KCA mutation and HRAS overexpression in R/M HNSCC benefit from the combination of alpelisib and tipfarnib.^150^ Copanlisib, a selective PI3K inhibitor, showed adverse toxicity and limited efficacy in a large number of patients with R/M HNSCC when combined with cetuximab.^151^

mTOR inhibitors include everolimus, temsirolimus, buparlisib, rapamycin, and CC‐115. Studies have shown that everolimus is ineffective alone or in combination with erotinib in unselected patients with R/M HNSCC.^152^, ^153^ Recently, a clinical study found that everolimus combined with CT for HNSCC was well tolerated, with an overall response rate of 75.6% and tumor size reduction of ≥50% in 20 patients.^154^ Unlike everolimus, temsirolimus was poorly tolerated when combined with erlotinib.^155^ However, temsirolimus monotherapy significantly reduced pS6 and p4E‐BP1 in tumors, as well as pS6 and pAKT in PBMC with minimal and reversible side effects.^156^ CC‐115 is a novel dual DNA‐PK and TOR kinase inhibitor, and the results of clinical trials (NCT01353625) indicate that CC‐115 is well tolerated and is considered as a promising new anticancer agent.^157^

AKT inhibitors such as MK‐2206 (NCT01349933), ipatasertib (NCT05172245), BYL719 (NCT02145312), and perifosine (NCT00062387) are under clinical trials. Currently, more clinical research are needed to demonstrate the efficacy of AKT inhibitors in HNSCC.^2^

#### 3.1.3 Targeting JAK/STAT

JAK/STAT pathway inhibitors like JAK1/2 inhibitor (ruxolitinib and AZD1480) and STAT3 inhibitor (danvatirsen and TTI‐101). Ruxolitinib and AZD1480 have shown good antitumor activity in PDX models associated with HNSCC. A clinical trial (NCT03153982) of ruxolitinib in patients with HNSCC is currently underway. AZD1480 showed antitumor efficacy in PDX models, which may provide a new prospect for HNSCC treatment.^55^ One phase Ib/II clinical trial (NCT02499328) showed that the treatment of danvatirsen combined with durvalumab was more effective than PD‐L1 treatment in R/M HNSCC patients.^158^ TTI‐101 has exhibited antitumor activity in many preclinical cancer models, which could inhibit tumor growth and reverse liver damage and fibrosis, and a clinical trial (NCT05668949) is underway to examine its effects.

#### 3.1.4 Targeting NF‐κB

Inhibition of the IKKβ/NF‐κB signaling pathway can improve treatment efficacy in cisplatin‐resistant HNSCC.^159^, ^160^ Here, we mainly reviewed two NF‐κB inhibitors (bortezomib and triptolide).

Bortezomil is a proteasome inhibitor targeting 26S proteasome and inhibit the activation of NF‐κB. The literature suggests that bortezomib may inhibit tumor growth in combination with RT in HNSCC.^159^ Du et al.^161^ showed that bortezomib combined with dasatinib may be effective in cisplatin‐resistant HNSCC patients. Several phase I studies combining Bortezomib with radiation therapy (NCT01445405, NCT00329589, NCT00629226, and NCT00011778) are currently conducting. Preclinical and clinical data suggest that the combination treatment may be promising; however, further mechanistic studies are still needed. Another NF‐κB inhibitor triptolide has immunosuppressive, anti‐inflammatory, and antiproliferative effects. Preclinical studies have shown that triptolide and minnelide can induce apoptosis by activating wild‐type p53. A clinical trial (NCT05791136) is evaluating its efficacy when combination with radiation therapy for ESCC.^162^

#### 3.1.5 New targeting strategies

Emerging studies have shown that signaling pathways such as HER2,^163^ HER3, ALK1, MEK, RAF, CDK, IDO1, CCR4, and Nectin 4 can also be used as targets for HNSCC clinical trials. Trastuzumab selectively binds to HER2 with high affinity.^164^ Numerous studies have investigated the use of CDX‐3379, ISU104, and duligotuzumab targeting HER3 in the treatment of HNSCC. Notably, KTN3379 has been shown to significantly inhibit the proliferation of HPV‐positive HNSCC.^165^ Patritumab is a fully human McAb target HER3, a phase Ib study (NCT02633800) showed that the combination strategy of patritumab, cetuximab, and platinum did not improve PFS and OS, and all patients had more than one adverse event, resulting in early termination of the trial.^166^

### 3.2 Immunotherapy

**FIGURE 5:**
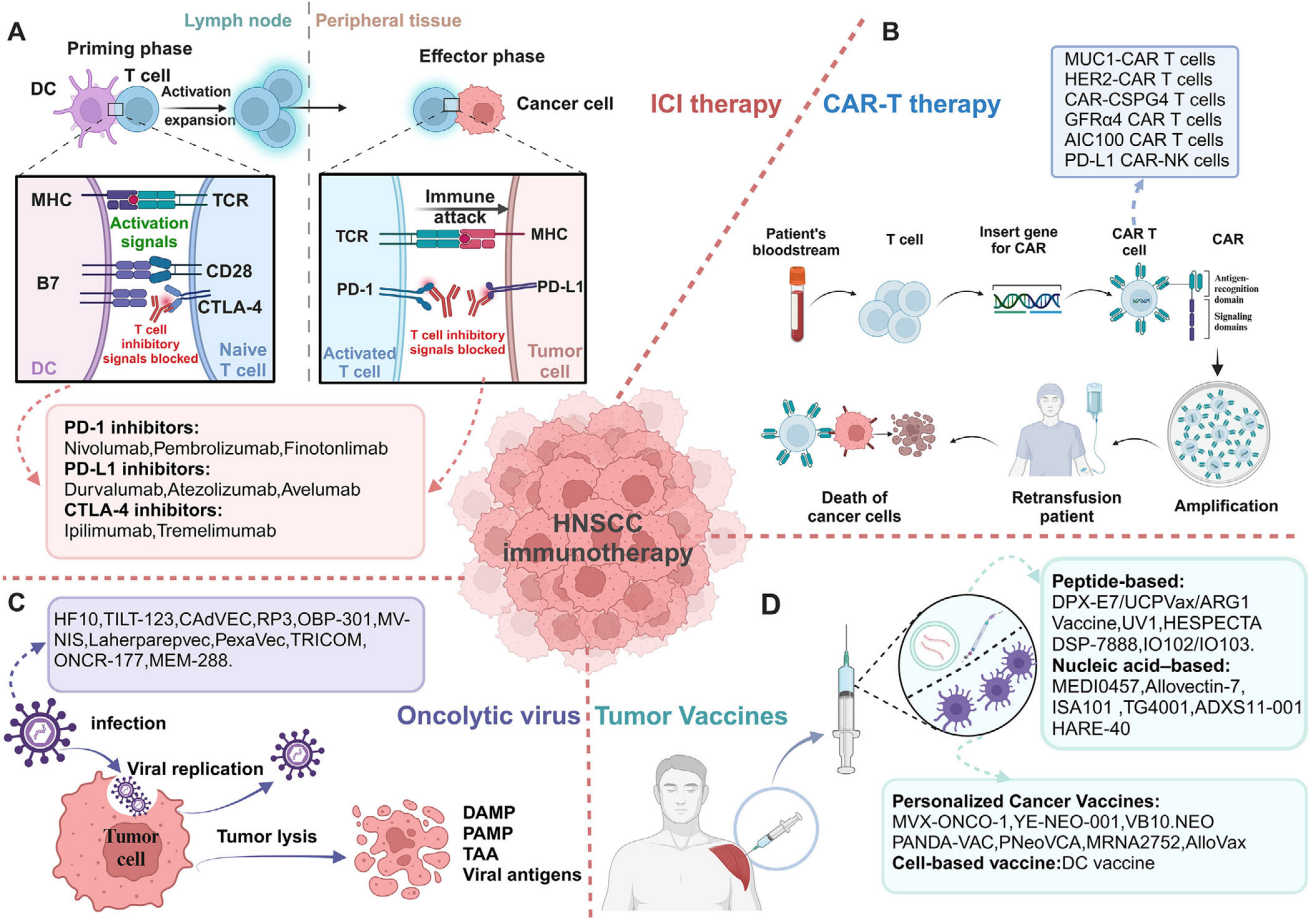
Immunotherapy strategies for HNC. Clinical immunotherapy strategies for HNC take various forms: (A) Immunotherapies target various coinhibitory (PD‐1/PD‐L1, CTLA‐4/B7) and costimulatory (TLR, OX40) signaling molecules present on the surface of immune cells. The application of ICIs and costimulatory agonists can achieve effective antitumor immune responses. (B) CAR‐T therapy involves the extraction of tumor‐specific T cells from patients, followed by in vitro amplification and reinfusion therapy. (C) OV therapy exerts antitumor effects through the direct killing of tumor cells by the virus. (D) Antigenic vaccines are a form of active immunotherapy in which the antigen is derived from the tumor. These vaccines can induce antitumor immune responses against TAAs and TSAs. TAAs, tumor‐associated antigens; TSAs, tumor‐specific antigens (created with BioRender.com).

HNC features one of the most inflammatory TMEs among solid tumors,^167^ making it highly receptive to immunotherapy.^168^ Current strategies include ICIs, adoptive cell therapies, oncolytic virus (OV) therapies, cancer vaccines, and immunomodulators^133^, ^168^ (Figure 5).

#### 3.2.1 Targeting PD‐1/PD‐L1

Nivolumab, the first US FDA‐approved anti‐PD‐1 McAb for R/M HNSCC, has shown a significant therapeutic effect in platinum‐sensitive R/M HNSCC patients.^169^, ^170^, ^171^, ^172^ Pembrolizumab, another highly selective McAb with high affinity for PD‐1^132^, has also demonstrated good safety and efficacy in various clinical trials.^173^ Pembrolizumab combined with platinum and 5‐fluorouracil is an appropriate first‐line treatment for R/M HNSCC.^174^ Finotonlimab is an innovative recombinant human McAb against PD‐1, which can specifically bind to PD‐1, enhance the function of CD8^+^ T cells, and inhibit tumor growth. Four clinical trials of finotonlimab are in progress for R/M HNSCC (NCT04146181, NCT05552807, NCT04146402, NCT04146181).

Durvalumab is a high‐affinity humanized McAb targeting PD‐L1, restoring the immune response and killing tumor cells.^175^ Durvalumab showed significant efficacy and good safety in a phase I/II clinical trial (NCT01693562). Monotherapy of duvazumab showed good antitumor activity and safety in R/M HNSCC patients with PD‐L1 overexpression (NCT02207530). However, neither Durvalumab alone or in combination with temlimumab significantly alleviate patients' OS (NCT02369874). Atezolizumab and avelumab are two novel PD‐L1 inhibitors that have shown good activities in multiple clinical trials. Other clinical studies showed that patients with HPV+ HNSCC benefited more from immunotherapy (ORR: 26.5% in the HPV‐positive group vs. 7.9% in the HPV‐negative group)^176^ (Figure 5A). Recent research showed that PD‐L1 expression is positively correlated with OS when treated with PD‐1 or PD‐L1 inhibitors.^177^

#### 3.2.2 CTLA‐4 antibodies

Ipilimumab and tremelimumab are mono‐antibodies, which block CTLA‐4 and are currently being tested for efficacy in HNSCC, despite promising results in other tumors.^133^ Wang et al.^178^ found that blocking the CTLA‐4 receptor can activate CD8^+^ T cells, increase production of IFN‐γ and TNF‐α, activate the STAT1/IRF1 axis and trigger tumor cell heat death in HNSCC.

Furthermore, several antibodies and small molecules targeting other immune checkpoints are still in clinical development, such as LAG3, TIGIT, TIM3, B7H3, CD39, CD73, adenosine A2A receptors, and CD47.^179^ However, due to its shortcomings such as limited efficacy of single drug, large side effects and use plan to be optimized, it is mainly combined with PD‐1 antibody to amplify the tumor inhibition effect of the latter. First‐line nivolumab and ipilimumab was evaluated for the treatment of R/M HNSCC (NCT02741570). The results showed that Nivolumab combined with ipilimumab showed a good safety profile^180^ (Figure 5A).

#### 3.2.3 New immunotherapy strategies

In addition to ICIs, new immunotherapy strategies include CAR‐T therapy, OVs therapy, tumor vaccines, and immunomodulators.

Immune evasion of cancer cells is mainly related with a lack of MHC‐associated antigen presentation in convention immunotherapy of HNSCC. CAR‐T therapy has significant advantages without MHC association.^181^ However, due to the high tumor heterogeneity, complex tumor structure, lacking of tumor‐specific antigens, TME immunosuppression, treatment‐related toxicity, and the risk of off‐target adverse events, the antitumor effect of CAR‐T cell therapy on HNSCC is weak, and the side effect is large, which is the obstacle of clinical transformation.^181^, ^182^ Recently studies have shown that selecting a suitable CAR target protein is of great significance in enhancing the killing ability of T cells. A series of trials of CAR‐T cells treatment have shown that MUC1, HER‐2, EGFR, CD70, LMP1, CD44v6, CD276 (B7‐H3), CD98hc, NKG2DL, FAP, HER3, and NKGD2 are promising CAR‐targeted proteins for HNSCC immunotherapy^180^ (Figure 5B).

OV therapy has great potential in local tumor control, low incidence, and safety, and it has become the fourth generation of tumor immunotherapy after ICI therapy.^183^ OV is a kind of naturally mutated double‐stranded enveloped DNA viruses or genetically engineered viruses, like adenovirus, vaccinia virus, reovirus, measles virus, herpes virus, parvovirus, coxsackie virus, VSV,^184^ and newcastle disease virus. OV exert toxic effects on tumor cells through multiple mechanisms including autolysis, immune cell homing, disruption of vascular supply, and enhancement of other adjuvant anticancer therapies.^175^, ^185^ Oncorine is the earliest OV used for HNC.^186^ Compared with conventional systemic CT, intratumoral injection of oncorine showed significant efficacy and good safety in a phase III clinical trial.^133^, ^186^ ONYX‐015, an adenovirus attenuated E1B gene, selectively inactivates the p53 gene. Intratumorally injection of ONYX‐015 improved survival of patients in a phase II clinical trial; however, the tumor is prone to rapid recurrence. And the phase III trial of ONYX‐015 is under study.^187^ Besides, many other therapeutic OVs are currently investigated in clinical trials, including oncolytic measles virus (MV‐NIS, NCT01846091) encoding the thyroid sodium iodide transporter, oncolytic herpesvirus carrying GM‐CSF (T‐VEC, NCT02626000), recombinant fowlpox virus expressing B7.1, ICAM‐1, LFA‐3, CEA, MUC1 (TRICOM, NCT00021424), and dioncolytic adenovirus (VISTA, NCT03740256).^133^ When used in combination with other immunotherapies, such as ICIs, CARs, and autologous tumor infiltrating lymphocytes, OV therapy significantly improved treatment for a range of tumor types, and reduced side effects and resistance, including HNSCC^184^ (Figure 5C).

With the emergence of tumor neoantigens, tumor vaccine therapies have broadened the immunotherapy landscape for patients with R/M HNSCC. DCs as antigen delivery carriers are a new focus in the development of HNSCC cancer vaccines.^188^ Current DC‐based vaccination approaches mainly rely on in vitro production of monocytes or CD34^+^ cell‐derived DCS loaded with antigens, which can be activated with various TLR ligands and cytokines, then activated DCs can be reinjected into HNSCC patients to promote cytotoxic T cell responses.^175^ Schuler et al.^189^ reported a phase I clinical trial (NCT00404339) of a selected wild‐type p53 polypeptide vaccine based on autologous monocyte derived DC loading. The results showed that Treg levels continued to decline, and the 2‐year survival rate was 88%.^189^ Recently, a recent study reported a vaccine administered with Wilms Tumor 1 (WT1) peptide‐loaded DC. The results showed that no vaccine‐related serious adverse reactions, extended survival, and enhanced WT1‐specific immunity were observed in 11 patients with R/M HNSCC combined with conventional CT.^175^ Xu et al.^190^ also reported a hybrid nanovaccine (Hy‐M‐Exo) that inherits CCR7, a key protein of DCMV lymphatic homing, and shows higher LN targeting efficiency. At the same time, a robust T cell response was induced by retained tumor antigens and endogenous danger signals in the hybrid nanovaccine activated APCs and showed good therapeutic effect in HNSCC mouse model, which suggest a viable strategy for antitumor immunotherapy.^190^ In a completed phase 2 study with CPI‐experienced R/M HNSCC, FLX475a selective CCR4 antagonist in combination with pembrolizumab was shown to be well tolerated and has good efficacy particularly in those with HPV‐positive tumors.^191^ A neoantigen DC vaccine showed significant efficacy and good safety in a phase Ib clinical trial of ESCC (NCT 05023928).^192^ (Figure 5D).

Currently, immunomodulators widely studied include Toll‐like receptor agonists TLR8 agonist motolimod (VTX‐2337) and TLR9 agonists (EMD 1201081, SD‐101, CMP‐001), OX40 agonists (MEDI0562, INBRX‐106, BGB‐A445, PF‐04518600), and cytokine modulators (IL‐12, IRX‐2, NT‐I7, N‐803). In addition, HB‐201 and HB‐202, an arenavirus‐based immunotherapy, enhance antitumor immunity in HPV16^+^ HNSCC.^193^ Different subtypes of B cells and TLSs in the TME of HNSCC patients are revealed to impact the efficacy of ICIs.^194^ Therefore, new therapeutic strategies based on B cells can be developed to enhance immunotherapy responses in HNSCC.

### 3.3 Gene therapy

Gene therapy has been widely utilized for cancer treatment, significantly enhancing antitumor efficacy. Various gene therapy agents employing the rAd‐p53 vector have been used in cancer treatment, with clinical gene therapy programs in China using rAd‐p53 for HNC treatment since 1998. Oncorine, when combined with CT, has proven both safe and effective in Chinese clinical trials for HNSCC. Following oncorine, two additional oncolytic viral agents, H103 (developed by Shanghai Sunway Biotech Co., Ltd.) and KH901 (developed by Chengdu Kanghong Biotechnology Co., Ltd.), have also been explored in Chinese clinical trials.^186^

E10A, a human endostatin gene carried by a type‐5 recombinant replication‐deficient adenovirus vector developed by Guangzhou Double Bioproducts, Co., Ltd. (Guangzhou, China), can be directly introduced into tumor cells. Here, it is translated into endogenous endostatin protein to limit vascularization.^195^ Another similar adenovirus, EDS01, combined with CT for HNC, entered a multicenter randomized phase II clinical study in 2017. In the phase I clinical trial in China, intratumoral injection of EDS01 showed better therapeutic effects against metastatic tumors. This offers new insights into the treatment of advanced HNC.

Suicide gene therapy, such as adenoviral vector (AdV)‐TK, is another form of gene therapy. AdV‐TK is an engineered replication‐incompetent adenovirus vector containing a suicide gene called HSV‐TK. The protein product of HSV‐TK converts the nontoxic antiviral drug GCV into a highly cytotoxic phosphorylated form. The HSV‐TK/GCV system was adapted in a phase I Chinese clinical study. Intratumoral injection of AdV‐TK followed by systemic administration of GCV proved to be both safe and effective.^196^ Additionally, another clinical study in China demonstrated significant efficacy when combining AdV‐TK/GCV with photodynamic therapy for oral cancer.^197^

However, the success rates of AdV‐based gene therapy for HNSCC have not been satisfactory, likely due to low AdV transduction efficiency. To address this, scientists have developed mutated AdV vectors incorporating the integrin‐binding motif RGD, which have shown promising efficacy.^198^

Recently, a therapeutically relevant fusion transcript named UBE3C–LRP5 fusion has been identified in HNSCC. This represents a promising therapeutic target for HNC, and preliminary findings suggest that pyrvinium pamoate may effectively target this translocation.^199^

### 3.4 Epigenetic modification therapy

Epigenetic modifications play crucial role in the progression of HNC, and treatments targeting these modifications have shown great potential. Epigenetic drugs, including DNMT inhibitors and HDAC inhibitors, have been extensively researched in both preclinical and clinical HNC studies. Two DNMT inhibitors including azacytidine and decitabine are under clinical trials in HNC. Azacytidine can inhibit cell growth, induce cell death, downregulate the expression of HPV genes, and stabilize p53 and suppress the expression of MMPs in vitro and in vivo.^200^ Decitabine has been reported to reverse methylation and sensitive cisplatin in HPV‐negative HNSCC. The preclinical data suggest that HDAC inhibition could sensitize HPV‐negative HNSCC cells to cisplatin and suppress their proliferation, migration and invasive potential.^201^ HDAC inhibitors currently in clinical trials for HNSCC include romidepsin, panobinostat, vorinostat, valproic acid, abexinostat, and CUDC‐101.^202^ A trial investigating romidepsin (NCT00084682) as a monotherapy for R/M HNSCC showed limited clinical efficacy and tolerability.^203^ However, combining vorinostat with cisplatin/RT has shown good tolerability and encouraging efficacy in HPV‐negative HNSCC.^204^ Additionally, combining vorinostat with pembrolizumab has demonstrated higher response rates in advanced staged HNSCC.^204^

### 3.5 Combination therapy

HNC is a highly heterogeneous tumor, and the effectiveness of standalone treatments like surgery, radiation, or CT is often limited. In recent years, the advent of immunotherapy and targeted therapy has led to significant breakthroughs in treating R/M HNC.^205^ By searching the keywords “combination therapy of head and neck cancer” on ClinilcalTrials.gov, we found 2113 combined treatment strategies for HNC, including postoperative combined CT and McAb therapy, RT combined CT and CT combined McAb therapy, RT combined with targeted drug therapy, immunosuppressive therapy combined with CT, and so on.^206^ Among all relevant clinical trials, the research contents are shown in Table 3. Otherwise, a recent study showed that HSP90 inhibitor ganetespib could increase the therapeutic efficacy of RT for HNC.^207^ With the deepening of the pathogenesis of HNC, the advancement of precision medicine or the identification of biomarkers, such as HOXB9,^208^ iron death related gene CISD2,^209^ Hedgehog (HH),^210^ FUT6,^211^ cysteine‐rich protein 2 (CSRP2),^212^ homeobox‐D 1,^213^ variant transcription factor 5 (ETV5^214^), and the developing of neoadjuvant chemoimmunotherapy (NACI) and photoimmunotherapy,^215^ clinicians can develop personalized treatments and predict outcomes based on each patient's unique biochemical and genetic profile. This approach further broadens the scope of combination therapy available for HNC patients. For additional details on ongoing clinical trials, refer to Table 3.

**TABLE 3:** Clinical trials of combined treatment strategies for HNCs.

Combinations	Interventions	Target (s)	Clinical trials	Phase	Enrollment	Indication
ICIs + TriAd vaccine	M7824, N803, TriAd vaccine (ETBX‐011, ETBX‐051 & ETBX‐061)	PD‐L1, TGF‐β MUC1, IL‐15	NCT04247282	I/II	21 (Actual)	Head and neck neoplasms, HNC
ICIs + Anti‐B7‐H3 antibody	Enoblituzumab, retifanlimab, tebotelimab	PD‐1, LAG3, CD276	NCT04634825	II	62 (Actual)	Head and neck neoplasms, HNC, HNSCC
ICIs + PI3K inhibitor	Duvelisib, pembrolizumab	PI3Kγ, PI3Kδ	NCT04193293	I/II	2 (Actual)	HNSCC
ICIs + AKT inhibitor	Ipatasertib pembrolizumab	AKT, PD‐1	NCT05172258	II	52 (Estimated)	HNSCC, R/M HNSCC, R/M OCSCC, R/M OSCC, R/M LSCC
ICIs + ATR inhibitor	Elimusertib pembrolizumab	ATR, PD‐1	NCT04576091	I	37 (Estimated)	HNSCC, R/M HNSCC, R/M OCSCC, R/M OSCC, R/M LSCC, recurrent salivary gland carcinoma
ICIs + Oncolytic virus	OBP‐301, pembrolizumab	Telomerase, PD‐1	NCT04685499	II	1 (Actual)	HNSCC with inoperable recurrent or progressive disease
ICIs + Radiation	Pembrolizumab, radiation	PD‐1	NCT04318717	II	16 (Estimated)	MMHN
	Nivolumab, radiation	PD‐1	NCT03758729	II	26 (Estimated)	Locally advanced, unresectable MMHN
	Ipilimumab, nivolumab, radiation	PD‐1, CTLA‐4	NCT03799445	II	180 (Estimated)	HPV‐mediated (p16‐Positive) OC, HPV+ OSCC, oropharyngeal basaloid carcinoma, OTSCC, soft palate squamous cell carcinoma
ICIs+radiation +chemotherapy	Carboplatin, cisplatin, fluorouracil, paclitaxel, pembrolizumab, radiation	PD‐1, DNA	NCT05721755	III	290 (Estimated)	HPV‐mediated (p16+) OC, metastatic HNSCC, metastatic LSCC, metastatic OCSCC, metastatic OSCC
ICIs + IL‐2R agonists + radiation	Pembrolizumab, NKTR‐214, palliative radiation	IL‐2R	NCT04936841	II	5 (Actual)	HNC
ICIs+chemotherapy	Carboplatin, cemiplimab, paclitaxel	PD‐L1	NCT04862650	II	42 (Estimated)	HPV‐mediated (p16+) OC, R/M HNSCC, R/M LSCC
ICIs + EGFR inhibitor + chemotherapy	Atezolizumab, bevacizumab, carboplatin, cisplatin, Cetuximab, docetaxel	EGFR, PD‐L1, DNA	NCT05063552	II/III	430 (Estimated)	R/M HNSCC, R/M NSCC, R/M LSCC R/M lip and oral cavity carcinoma R/M NCSCC
EGFR inhibitor + radiation + chemotherapy	cetuximab, cisplatin, radiation	EGFR	NCT01855451	III	189 (Actual)	HPV‐positive OSCC

Abbreviations: HNC, head and neck cancer; HNSCC, head and neck squamous cell carcinoma; LSCC, laryngeal squamous cell cancer; MMHN, mucosal melanoma of the head and neck; MMHN, head and neck mucosal melanoma; NSCC, nasopharyngeal squamous cell carcinoma; OC, oral cavity; OCSCC, oral cavity squamous cell carcinoma; OSCC, oral squamous cell carcinomas; OTSCC, oral tongue squamous cell carcinoma; OCSCC, oral cavity squamous cell carcinoma.

*Sources*: Data were obtained from ClinicalTrials.gov.

## 4 CONCLUSIONS AND PROSPECTS

Over the past three decades, despite advancements in understanding the pathogenesis, development of new detection methods, and the emergence of innovative targeted therapies and immunotherapies, challenges such as drug resistance and tumor recurrence or metastasis continue to complicate the treatment of HNC.^13^, ^216^ This comprehensive review delves into the innovative pathogenesis of HNC and summarizes various therapeutic strategies including molecular targeted therapy, immunotherapy, gene therapy, and epigenetic modifications for HNC.

Targeted therapy of HGF/MET may require a combination of EGFR inhibitor strategies.^60^ Studies have showed that novel combinations of inhibitors targeting EGFR, HER2, and c‐Met are more effective against relapsed and resistant HNSCC compared with targeting these pathways individually.^217^ Related studies have shown that signaling pathways such as HER2118, HER3, ALK1, MEK, RAF, CDK, and IDO1 can also be used as targets for HNSCC clinical trials. Although ICIs therapy has improved outcomes for various cancers, only a small percentage of patients achieve a lasting response. About 20−30% of patients still develop primary or secondary resistance, resulting in tumor recurrence and metastasis.^179^ Factors such as PD‐L1 expression, HPV status, tumor mutational burden, and interferon levels also influence the effectiveness of immunotherapy. Therefore, combining ICIs with other strategies is necessary to combat tumor immune escape.^218^ ICIs have been reported to improve sensitivity to salvage CT. As the tumor immune microenvironment changes after ICI administration, markers like the neutrophil‐to‐lymphocyte ratio and CRP levels can predict the efficacy of the treatment.^219^ Novel agents targeting immune checkpoints and costimulatory receptors, such as LAG‐3, OX40, HLA‐E, and 4‐1BB, are currently evaluated in clinical trials for HNSCC. Additionally, combining ICIs with other anticancer strategies like radiation, surgery, and CT is advancing rapidly.^168^ In a phase II trial (NCT03799445), the combination therapy of anti‐PD‐1 and anti‐CTLA‐4 antibodies with RT is being investigated for patients with HPV‐positive oral squamous cell carcinoma. HPV‐associated HNSCC has shown greater sensitivity to radioimmunotherapy due to its unique genetic and immunogenomic landscape.^220^ CAR‐T therapy in solid tumors, while promising, still faces numerous challenges that need to be addressed to benefit more HNSCC patients.

Despite these advancements, the OS rate for HNC patients remains low. There is a need to discover new prognostic targets and develop new targeted therapies, such as antibody–drug conjugates (ADCs), photodynamic therapy, radionuclide therapy, and mRNA vaccines, to enhance efficacy and minimize side effects. Clinical trials are ongoing for ADCs like ADRX‐0706 (NCT06036121) and BAT8007 (NCT05879627).^221^ In 2020, Japan approved the photoimmunotherapy drug Akalux (ASP‐1929, cetuximab conjugated with IRDye700DX) for use with laser system medical devices in treating unresectable locally advanced or recurrent HNC. Iopofosine I‐131 (CLR 131), a novel targeted small molecular phospholipid ether drug conjugate, demonstrated good safety in a phase I study.^222^ mRNA cancer vaccines like Lipo‐MERIT, encoding a fixed combination of shared cancer antigens for HNC, (BNT113/NCT04534205) are also in clinical trials.^223^

In the near future, mapping the specific genetic profiles of HNC will aid in discovering biomarkers for prognosis. Moreover, highly efficient and strongly immunogenic vaccines targeting neoantigens are rapidly developing.

## AUTHOR CONTRIBUTIONS

Yan Liu performed the clinical trial retrieval, main text writing, and production of some figures and tables. Nannan Zhang contributed with the introduction writing and full text modification and supervision. Yi Wen performed the searching of clinical trials and adjusting article format. Jiaolin Wen contributed with the supplement and enhancement of main text writing, production of some figures and tables, and payment for publishing fee. All the authors have read and approved the final manuscript.

## CONFLICT OF INTEREST STATEMENT

No potential conflict of interest were disclosed.

## ETHICS STATEMENT

Not applicable.

## Data Availability

Not applicable.
